# Synthesis of High Surface Area α-K_y_MnO_2_ Nanoneedles Using Extract of Broccoli as Bioactive Reducing Agent and Application in Lithium Battery

**DOI:** 10.3390/ma13061269

**Published:** 2020-03-11

**Authors:** Ahmed M. Hashem, Hanaa M. Abuzeid, Martin Winter, Jie Li, Christian M. Julien

**Affiliations:** 1 Inorganic Chemistry Department, National Research Centre, 33 El Bohouth Str. (former El Tahir Str.), Dokki-Giza 12622, Egypt; ahmedh242@yahoo.com; 2 Institute of Energy and Climate Research, Helmholtz-Institute Muenster (HI MS), IEK-12, Forschungszentrum Juelich GmbH, Corrensstr. 46, D-48149 Muenster, Germany; hanaa20619@hotmail.com (H.M.A.); martin.winter@uni-muenster.de (M.W.); jie.li@uni-muenster.de (J.L.); 3 MEET Battery Research Center, Institute of Physical Chemistry, University of Muenster, Corrensstr. 46, D-48149 Muenster, Germany; 4 Institut de Minéralogie, Physique des Matériaux et Cosmologie (IMPMC), Sorbonne Université, CNRS-UMR 7590, 4 place Jussieu, 75252 Paris, France

**Keywords:** α-K_y_MnO_2_, nanoneedles, lithium-ion batteries, green synthesis, cathode

## Abstract

With the aim to reduce the entire cost of lithium-ion batteries and to diminish the environmental impact, the extract of broccoli is used as a strong benign reducing agent for potassium permanganate to synthesize α-K_y_MnO_2_ cathode material with pure nanostructured phase. Material purity is confirmed by X-ray powder diffraction and thermogravimetric analyses. Images of transmission electron microscopy show samples with a spider-net shape consisting of very fine interconnected nanoneedles. The nanostructure is characterized by crystallite of 4.4 nm in diameter and large surface area of 160.7 m^2^ g^−1^. The material delivers an initial capacity of 211 mAh g^−1^ with high Coulombic efficiency of 99% and 82% capacity retention after 100 cycles. Thus, α-K_y_MnO_2_ synthesized via a green process exhibits very promising electrochemical performance in terms of initial capacity, cycling stability and rate capability.

## 1. Introduction

Lithium-ion batteries (LiBs) have been widely used to power many systems (e.g., portable electronics, tools, hybrid and electric vehicles, etc.) [1,2,3]. However, to increase the gravimetric energy density, it is necessary to find optimized, safe and suitable electrode materials, which are the limiting elements of LiBs [4,5].

Manganese dioxide (MDO) is an abundant, rather cheap and green material, which is not only used in primary lithium batteries [6], but also developed as advanced electrode material for Li-ion, Li-sulfur or Li-air secondary batteries [7,8,9,10,11,12,13]. It can deliver a theoretical capacity of ~320 mAh g^−1^ based on the transfer of one electron per formula in the potential range of 1.5–4.0 V (vs. Li^+^/Li). In addition to applications in batteries, MnO_2_ has also been used as a component in composite electrodes for electrochemical supercapacitors [14]. It crystallizes in various polymorphic forms, e.g., α-, β-, γ-, λ- and δ-MnO_2_, etc., which differ from each other by the different assembly of octahedral MnO_6_ units. Thus, the electrochemical performance of MnO_2_ strongly depend on the number of sites available for Li-ion insertion in its tunnel framework. According to different conditions of reaction or synthesis methods, MnO_2_ can be obtained with various morphologies (e.g., nanowires (NWs), nanoneedles (NNs), nanofibers (NFs), nanoflakes (NKs), nanosheets (NSs), nanorods (NRs), etc.) that promote suitable electrode material for LiBs [10]. According to intensive investigations on nanostructured materials, the needle-shaped specimen is proved to be a good choice in order to enhance the electrochemical performance of MnO_2_ [15]. The nanoscale and nanofiber-type morphology can provide more active sites for Faradaic reaction and, hence, reduce the diffusion time of lithium ions in the core of particles due to the one-dimensional transport pathway and the large surface-to-volume ratio [16].

Among different MnO_2_ polymorphs, the tetragonal α-phase has attracted special attention because its large 2 × 2 tunnels are favorable for the transportation and accommodation of foreign ions [17,18], such as K^+^ in the cryptomelane-type K_x_Mn_8_O_16_ compounds [19]. Zhang et al. reported that K_0.25_Mn_2_O_4_ nanofibers exhibit excellent lithium insertion properties with high charge capacities and good rate capability [20].

**Figure 1:**
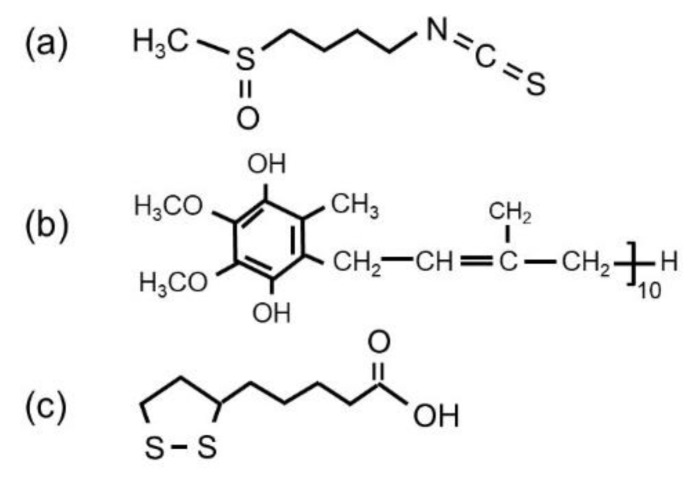
Schematic representation of the chemical composition of broccoli extract. Chemical structure of (**a**) sulforaphane, (**b**) vitamin coenzyme Q10 and (**c**) α-lipoic acid.

The preparation of α-MnO_2_ nanostructured particles has been realized by different techniques, including co-precipitation [16], comproportionation [21], hydrothermal [22], sonochemical reduction [23], micro-emulsion route [24], simple redox reaction [25] and reflux method [26]. Green synthesis is also one of these approaches using extracts of plants as reducing agents taking into account the presence of flavonoids and polyphenols [27,28,29]. Phenolic compounds are characterized to have at least one aromatic ring attached to one or more hydroxyl groups, with various arrangements of their carbon atoms. The reducing or antioxidant ability of flavonoids and phenolic acids is closely related to the number and position of hydroxyl groups in the molecule. The higher the number of hydroxyl groups, the higher the antioxidant activity [30]. Previously, we reported successful preparations of nanostructured MnO_2_ via biological reducing agents such as extract of green and black tea [27], citrus peel [31], and extract of orange peel [25]. Broccoli is a kind of vegetable similar to cauliflower and cabbage, all of them are rich in natural antioxidants, antimicrobial and anticancer activities [32]. Such vegetables have a high number of phenolic components and are appreciable sources of polyphenols, especially flavonoids [33]. Figure 1 shows a schematic representation of the chemical composition of broccoli extract including the antioxidant species (i.e., α-lipoic acid, sulforaphane and coenzyme Q10) [32,34]. These flavonoids and phenolic acids are present in leaves, flowering tissues and other parts of the broccoli plant.

The aim of this work is to prepare α-K_y_MnO_2_ nanoneedles with a low concentration of potassium ions using a two-step green synthesis approach with the extract of broccoli plant as reducing agent, and to investigate the electrochemical performance of the as-prepared sample. The structure and morphology of the prepared sample are investigated by X-ray diffraction (XRD), thermal gravimetric analysis (TGA), Brunauer–Emmett–Teller (BET) specific surface area method, and transmission electron microscopy (TEM). Cyclic voltammetry (CV) and galvanostatic charge-discharge (GCD) experiments are carried out in lithium-metal cells with α-K_y_MnO_2_ as the positive electrode. Finally, the rate capability and cyclability of α-K_y_MnO_2_ NNs are further investigated.

## 2. Materials and Methods 

All chemicals were analytical grade and used without further purifying. The redox reaction between KMnO_4_ (oxidizing agent) and the extract of broccoli (reducing agent) was directly employed to prepare K_y_MnO_2_ nanowires without templates or surfactants, thus the synthesis can be considered as a green approach. A concentrated extract of broccoli sprouts was obtained through fresh seasonal vegetables cleaned in distilled water followed by 10 min boiling at 100 °C [35]. After filtration of the decoction, the bioactive compound (2 g) was added to 3 g of KMnO_4_ (19 mmol) dissolved in 100 mL distilled water (DI) and acidified with 2 mL of 2.5 mol L^−1^ H_2_SO_4_. The entire mixture was stirred vigorously for 1 h at room temperature. Then, a change in color from purple to black was observed as KMnO_4_ was completely reduced by the broccoli extract and black precipitate was formed. The precipitate was isolated by filtration and washed several times by distilled water, then was dried overnight at 90 °C and further calcined at 300 °C for 5 h in air.

The crystal structure of the final product was determined by XRD using Philips X’Pert apparatus (Philips, Hambourg, Germany) equipped with a CuK_α_ X-ray source (λ = 1.54056 Å) in the 2*θ* range of 10–80°. TGA measurements were carried out using a thermal gravimetric analyzer (Perkin Elmer, TGA 7 series, Baesweiler, Germany) in the temperature range of 50–1000 °C at a heating rate of 10 °C min^−1^ in air. The morphology of the prepared sample was investigated by transmission electron microscopy (TEM, JEOL, JEM-2100, Electron microscope, Tokyo, Japan). The BET specific surface area was measured by nitrogen adsorption/desorption at 77 K in a relative pressure *P*/*P*_0_ = 0.0–1.0, with *P* and *P*_0_ being the equilibrium and saturation pressure, respectively, using a Quantachrome NOVA Automated Gas Sorption analyzer (Anton Paar GmbH, Blankenfelde-Mahlow, Germany). The pore size distribution and pore volume were estimated using the Barrett–Joyner–Halenda (BJH) method [36].

Electrodes for electrochemical testing were prepared by casting a slurry (N-methyl-2-pyrrolidone (NMP) used as the processing solvent) composed with 85 wt.% α-K_y_MnO_2_ active material, 10 wt.% conductive carbon C65 (TIMCAL), and 5 wt.% polyvinylidene fluoride (PVdF) onto an Al foil. The dried sheet was punched into ∅ 12 mm discs, and the mass loading of active material was evaluated to be 1.2–1.3 mg cm^−2^. These positive electrodes were assembled inside 2016 coin cells with Li metal foil as the counter electrode and 1 mol L^−1^ LiPF_6_ in ethylene carbonate:dimethyl carbonate (EC:DMC, 1:1 in vol.) as electrolyte. Cyclic voltammetry measurements were carried out at scan rate of 0.2 mV s^−1^ using a coin-cell with a 2-electrode configuration, that implies cumulative electrochemical response of both electrodes. The cycling and rate performance were obtained by galvanostatically discharging and charging cells on a Maccor series 4000 battery tester (Maccor Inc., Tulsa, OK, USA) between 4.0 and 1.5 V at 25 °C under different current densities from 0.1 to 10 C rate (1 C = 320 mAh g^−1^). 

## 3. Results

### 3.1. Morphology, Structure and Composition

The structure, composition and morphology of the as-prepared K_y_MnO_2_ were investigated by XRD, TGA, BET and TEM measurements. Particular attention was paid to characterize the crystal-chemistry of K_y_MnO_2_ synthesized via the reduction of potassium permanganate, which always induces a residual fraction of K^+^ ions (^VIII^K ionic radius of 1.51 Å) located in the 2 × 2 tunnels of the α-MnO_2_ lattice, whose main functions are templating and stabilizing the tunneled network [37]. Although numerous works published in the literature neglect this aspect. Several articles considered the presence of potassium as the combined outcome of the preparation of cryptomelane or birnessite MnO_2_ phases [27,38,39,40]. As a result, mixed +4 and +3 oxidation states of Mn cations could be induced in the α-MnO_2_ (Mn_8_O_16_) lattice (i.e., KMn^IV^_7_Mn^III^O_16_). Thus, the degree of insertion of K^+^ ions located in the crystal tunnels depends on the synthesis process as it will be discussed in infra.

**Figure 2:**
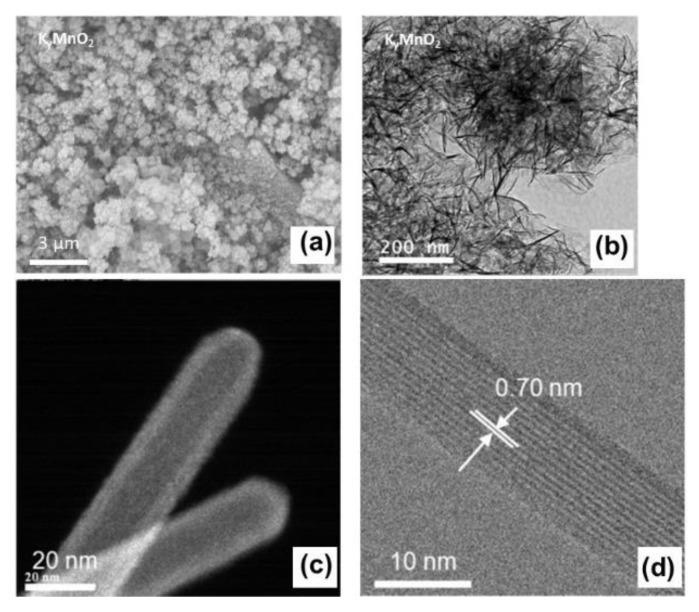
(**a**) SEM images of cryptomelane-type K_y_MnO_2_ nanoneedles, (**b**) TEM image showing the interconnected nanoneedles and (**c**,**d**) HRTEM images. The lattice fringes (image (**c**)) show an interplanar distance of 0.70 nm, which corresponds to the separation between the (110) crystal planes in the cryptomelane α-MnO_2_ structure.

SEM and TEM images of K_y_MnO_2_ are shown in Figure 2a–d. In the SEM picture (Figure 2a), micron-sized particles with average size ~500 nm have almost identical shape and distribution. The sample calcined at 300 °C for 5 h in air grows with the morphology of secondary particles (agglomerates) composed of interconnected nanoneedles. Particles are constituted by a complex arrangement of very fine interconnected nanoneedles with diameters in the range of 5–20 nm and lengths of 100–300 nm. Thus, K_y_MnO_2_ architecture is an assembly of nanoneedles interwoven into a mesoporous network as it will be characterized in infra. The HRTEM bright-field image of a selected well-crystallized nanoneedle (Figure 2d) displays uniform lattice fringes with an interplanar distance of 0.70 nm, which corresponds to the separation between the (110) crystal planes of the α-MnO_2_ phase indicating the well-ordered tunnel structure along the *b*-axis. These images suggest that the shape and small size of nanoparticles can be attributed to the fast reduction process of KMnO_4_ [16] and the acidification by 2.5 mol L^−1^ H_2_SO_4_. The dependence of chemical composition, the nanoparticle size and lattice structure with growing conditions (i.e., temperature and acidity) has been investigated in previous works [41,42,43,44,45,46]. For example, the cryptomelane phase is formed in an acidic solution, while the birnessite-type MnO_2_ is synthesized in a basic solution. Portehault at al. obtained cryptomelane nanowires (15–40 nm in diameter) when the pH value of the solution has been controlled in the range of 0.9–1.25 [21]. It appears that up-scalable synthesis can be achieved using a facile method at low temperature with very cheap green chemistry. Villegas et al. showed that the reduction of KMnO_4_ by H_2_O_2_ under acidic condition produced NNs with width of a few tens of nanometers [45]. In contrast, Kumar et al. addressed the preparation of highly-dispersed α-MnO_2_ NNs (20–30 nm in size) without K^+^ inclusion using a sonochemical hydrolysis of Mn(3)acetate with a solution close to neutral pH followed by mild drying [46]. 

**Figure 3:**
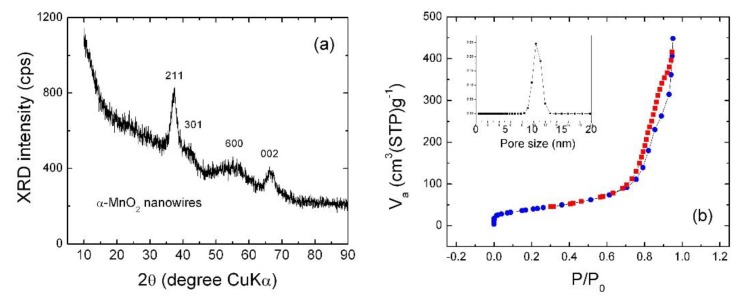
(**a**) XRD patterns of α-K_y_MnO_2_ nanoneedles. (**b**) N_2_ adsorption-desorption isotherm of α-K_y_MnO_2_ nanoneedles and the pore size distribution (inset).

Figure 3a shows the XRD pattern of as-prepared K_y_MnO_2_ NNs. The main characteristic peak appearing at 2*θ* value of 37° is indexed to the (211) plane, while other diffraction peaks at 42.0°, 55.5° and 66.4° with low intensity in pattern are assigned to the (301), (600) and (002) planes, respectively, of the tetragonal α-MnO_2_ phase (space group *I*4/*m*, JCPDS 44-0141) with no apparent impurities. No diffraction pattern attributable to other crystallographic MnO_2_ is observed. The low intensity is due to the poor crystallinity and the peak broadening indicates the nanosized character of α-K_y_MnO_2_ samples. The refined lattice parameters are *a* = 9.95(2) Å, *c* = 2.76(7) Å and *V* = 274.1 Å^3^. A comparison with values of the α-MnO_2_ crystal (JCPDS card 44-0141, *a* = 9.7845 Å, *c* = 2.8630 Å) confirms the lattice expansion along the *a*-axis direction, which may have originated by the presence of bigger six-coordinated Mn^3+^ ions (high-spin state, r_Mn_^3+^ = 0.645 Å) than Mn^4+^ (r_Mn_^4+^ = 0.53 Å). Meanwhile, the elementary unit volume matches well with values of the α-K_y_MnO_2_ structure, in which a low concentration of K^+^ ions is inserted in the 2 × 2 tunnels (i.e., typically *y* < 4 in α-K_y_Mn_8_O_16_) [43]. For example, a slight bigger elementary volume of 279.6 Å^3^ was reported by Vicat et al. for K_1.33_Mn_8_O_16_ [19]. Scherrer crystallite size calculated from the half-width at half-maximum of the (211) diffraction line at 2*θ* ≈ 37.6° is found to be of 4.4 nm, showing the polycrystalline character of α-K_y_MnO_2_ NNs. The combination of XRD data and HRTEM image reveals that NNs are composed of a few aggregated crystallites. This result is comparable with those obtained from α-K_y_MnO_2_ nanoneedles prepared by the mild synthetic method using the extract of tea as reducing agent, in which XRD patterns are dominated by the (211) Bragg line and the crystallite size is found to be ~4 nm [27]. 

Figure 3b shows the N_2_ adsorption desorption isotherm and Barrett–Joyner–Halenda (BJH) pore-size distribution for as-prepared α-K_y_MnO_2_. It can be noticed that the isotherm curve displays an increase in the amount of adsorbed N_2_ with increase of the *P*/*P*_0_ value; the appearance of a hysteresis loop indicates the hierarchical mesoporous structure of α-K_y_MnO_2_ NNs. At *P*/*P*_0_ = 0.97, the volume of N_2_ adsorbed is found to be 450 cm^3^ g^−1^. The mesoporous nature of the as-prepared α-K_y_MnO_2_ is also illustrated by the graph in inset of Figure 3b, which was obtained according to BJH method. Suib et al. [47] have described the formation of mesoporosity by aggregation of MnO_2_ nanoparticles (nanorods or nanoneedles) along the lateral faces. The mesopore-size distribution in the range 1–20 nm displays a single peak centered at 10.4 nm, with total pore volume of 0.950 cm^3^ g^−1^. The α-K_y_MnO_2_ samples (powders) are composed of secondary particles (agglomerates), which are interconnected nanoneedles (primary particles) with diameters in the range of 5–20 nm and lengths of 100–300 nm. The mesopores (10 nm in size) correspond to the intraconnecting voids existing between randomly-packed nanoneedles [47].

The effects of the particle size and the surface area of the nanostructured MnO_2_ materials were studied extensively (see, for example, [47,48,49,50,51,52,53,54,55]) because the mesoporous structure plays a significant role in enhancing the active sites at electrode surface and improves the electrochemical properties sequentially. Xia et al. stated that the mesopore size for MnO_2_ decreases from 8 to 4 nm with the increase of the hydrothermal synthesis temperature [48]. Li et al. reported an average mesopore diameter of ~8 nm for mesoporous MnO_2_ with a BET surface area of 226 m^2^ g^−1^ [49] The BET surface area value of 161 m^2^ g^−1^ is greater than values reported for MnO_2_ prepared by different methods (e.g., microemulsion method (123 m^2^ g^−1^) [50], hydrothermal synthesis (150 m^2^ g^−1^) [51], silica templating combined with ion-exchange route (142 m^2^ g^−1^) [52], mild reaction [53] and exfoliation [49]).

Thus, the synthesis technique used in this work appears to be efficient in providing mesoporous pure α-phase K_y_MnO_2_ NNs with relatively small particles. The K-birnessite-type MnO_2_ hydrothermally synthesized at 160 °C for 30 min exhibits a narrow pore size distribution around 5.4 nm [54]. Regarding the HRTEM results, the pore size value describes cross-section of a single nano-needle. The value of 10 nm is in good agreement with the inner diameter measured by HRTEM. Similar results were reported for mesoporous α-MnO_2_ nanorods (NRs) synthesized by reduction of KMnO_4_ with a triblock polymer assisted by sonication (i.e., a pore volume of 1.0 cm^3^ g^−1^ but smaller pore diameter of 13.5 nm) [23]. The mesoporous MnO_2_ nanorods prepared by the solvent-free synthesis method show a unique pore-size distribution peak positioned at 16 nm [55]. 

**Figure 4:**
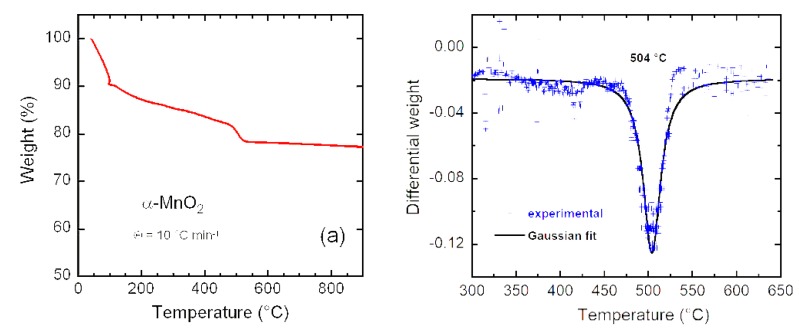
(**a**) TG curve of green synthesized K_y_MnO_2_ nanoneedles. (**b**) Differential weight d*W*/d*T* showing the decomposition temperature from α-K_y_MnO_2_ to Mn_2_O_3_.

The chemical composition of K_y_MnO_2_ NNs analyzed by inductively-coupled plasma (ICP) measurements shows 52.62 wt.% Mn and 1.45 wt.% K. This result confirms that the cryptomelane-type sample contains a small concentration of potassium. The formula can be calculated as α-K_0.03_MnO_2_, taking into account the error of measurements. The presence of K^+^ ions inside 2 × 2 tunnels is considered to be positive to stabilize the tetragonal structure and enhance the Li-diffusion kinetics [56]. This composition as well as the chemisorbed water were further determined by thermogravimetry (TG). The TG curve shown in Figure 4a displays three distinct weight losses. A first step of ~10% at ca. 120 °C (region I) is attributed to the loss of moisture from the sample surface. The second weight loss of ~20% in the range 200–400 °C (region II) is due to the removal of structural water. The third step (viewed as a fast drop) in the range of 480–520 °C (region III) is signed to the reduction of MnO_2_ to the non-conductive Mn_2_O_3_ (dense bixbyite structure) occurring with a release of oxygen (2MnO_2_→Mn_2_O_3_ + ½O_2_) via an exothermic reaction [57]. Several studies have shown that the onset temperature of the rapid weight loss in region III depends on the concentration of tunneled foreign ions (potassium or ammonium) in the cryptomelane K_y_Mn_8_O_16_ structure [39,58]. Generally, the MnO_2_→Mn_2_O_3_ transformation occurs in the temperature range of 480–670 °C, 480 °C being the decomposition temperature for a material without any large stabilizing cations and 670 °C being the transformation temperature of K_2_Mn_8_O_16_ to bixbyite [59]. The decomposition temperature of 504 °C was estimated from the differential (DTG) weight loss as shown in Figure 4b. Using different DTG behaviors of the literature, it was found that there is an almost linear relationship between the transition temperature and the content of K^+^ ions in α-K_x_Mn_8_O_16_. According to Figure 2 shown in [27], we can estimate a concentration of potassium (i.e., *x* = 0.035), which is close to the value obtained from ICP measurement. Thus, the amount of electrochemically inactive K^+^ ions (1.45 wt.%) is confirmed to be much smaller than that of Mn (52.82 wt.%), and does not obviously affect the specific capacity of the electrode.

### 3.2. Electrochemical Performance

**Figure 5:**
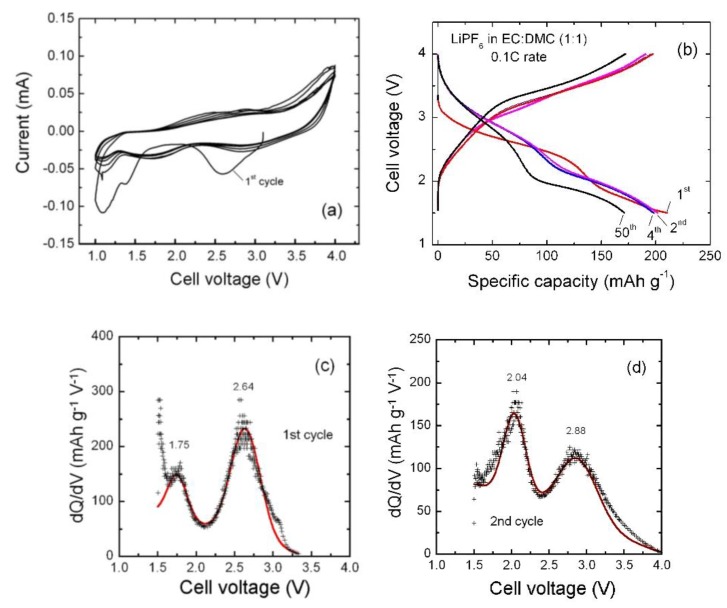
(**a**) Cyclic voltammograms of α-K_0.03_MnO_2_ nanoneedles recorded at scan rate of 0.1 mV s^−1^ in the voltage range from 1.0 to 4.0 V. (**b**) Voltage profiles of a K_0.03_MnO_2_ NNs‖Li cell over 50 cycles carried out at 30 mA g^−1^. (**c**,**d**) Incremental discharge capacity at the first and second cycles showing the electrochemical transformation after the first lithiation process.

The suitability of the α-K_y_MnO_2_ nanoneedles as a cathode material for lithium batteries was tested electrochemically by cyclic voltammetry (CV) and galvanostatic charge discharge (GCD) experiments using 2016-type coin cells. All measurements were carried out at room temperature. Note that without reference electrode, the choice of cell voltage range should exclude that of the lithium electrode. Figure 5a shows the CV curves of α-K_y_MnO_2_ NNs recorded at scan rate of 0.1 mV s^−1^ within the voltage range of 1.0–4.0 V. The redox peak is attributed to the Li insertion associated with the Mn^4+^/Mn^3+^ couple. The initial open circuit voltage (OCV) of the cell with α-K_y_MnO_2_ NNs electrode was 3.08 V. The broadening of CV peaks is attributed to the poor crystallinity of α-K_y_MnO_2_ electrode [27] rather than the nanosized morphology [60]. Two reduction peaks occur at about 2.58 and 1.37 V during the first discharge, which suggest multiple inequivalent sites for lithium insertion into MnO_2_ tunnels. The second cycle and subsequent ones differ notably from the first one with cathodic peaks shifted to higher voltage at 2.85 and 1.73 V. The voltage shift is very small for the anodic peak at the second cycle indicating a stabilization of the insertion/extraction mechanism in K_0.03_MnO_2_ host lattice. Poyraz et al. [39] demonstrated that cathodic-anodic peak positions and the peak separations depend on the K^+^ content in the tunnels. Sample with high K^+^ content have smaller initial discharge capacities compared to low K^+^ containing sample. The initial electrochemical modification (1^st^ cycle) is assigned to the “Li-cell formation” with the structural change that results from the so-called “loss of lithium inventory” [61]. A fraction of the Li^+^ ions inserted during the first discharge remains trapped in the tunnel structure. Both structural K^+^ and trapped Li_+_ ions stabilize the MnO_2_ framework for the second and further discharge-charge cycles. The inserted Li^+^ ions occupying the 8*h* and 8*h*’ Wyckoff sites of the tetragonal lattice requires different energies of formation [62].

However, during the first cycle, the cathodic peak shift has been observed by several workers, but the reason for such trend is not exactly clear [20,63,64]. The shift of the initial cathodic peak in K_0.25_MnO_2_ at about 2.22 V vs. Li^+^/Li toward higher potential in subsequent cycles was reported by Zhang et al. [20]. Some local restructuring or activation taking place during the initial Li insertion was conjectured. Two options can be considered. (i) As a part of Li is trapped in the K_y_MnO_2_ lattice, more Mn^3+^ ions remain at the end of the redox process. Mn^3+^ is a Jahn–Teller ion, which induce a lattice distortion and, consequently, a change in the Mn^4+/3+^ redox potential. (ii) The redox potential shift could be due to the reduction of Mn^3+^/Mn^2+^. Let us consider the standard potentials Mn^4+^/Mn^3+^ and Mn^3+^/Mn^2+^ redox reactions in solids. For aqueous systems, the Mn^IV^O_2_ → Mn^3+^ → Mn^2+^ redox reactions occur at the standard potential of 0.96 and 1.51 V vs. NHE, respectively. Mn^3+^/Mn^4+^ displays an oxidation potential at 0.8 V vs. RHE in perovskites. Spinel materials containing Mn^3+^ and Mn^2+^ also exhibit high activities at 0.8 V vs. RHE. The reduction of β-MnO_2_ and the formation of Mn_3_O_4_ (with tetrahedral Mn^2+^ and octahedral Mn^3^+) occurs at 0.70 V vs. RHE. For aprotic systems, the lithium pyrophosphate Li_2_MnP_2_O_7_ and LiMnPO_4_ olivine display a Mn^3+^/Mn^2+^ redox potential centered at 1.17 and 0.82 V vs. SCE, respectively. Wang et al. [65] reported the redox reactions between Mn species in MnO_2_: the anode and cathodic peaks at 0.45 and 0.22 V vs. SCE are assigned to the Mn^4+^/Mn^3+^ reaction, while 0.0 and −0.25 V are the anodic and cathodic peaks of the Mn^3+^/Mn^2+^ reaction. Thus, the difference in the cathodic peak positions of 0.47 V is higher than the potential shift (0.27 V) observed in our voltammograms.

The oxidation process occurs with the appearance of corresponding anodic peaks at 2.90 and 2.38 V. The overlapping of the charge–discharge profile of the second cycle with subsequent ones implies a good reversibility. Similar electrochemical features have been observed previously for K_0.25_MnO_2_ [20]. In addition, the peak separation voltage (polarization) between *E*_ox_ (2.85 V) and *E*_red_ (2.58 V) is Δ*E* = 0.27 V in the first cycle, but diminishes to as low as 0.05 V in forthcoming cycles, which is the other good evidence for high reversibility of as prepared α-K_y_MnO_2_. Furthermore, the good reversibility is due to the presence of K^+^ ions within the 2 × 2 tunnels of the α-MnO_2_ structure maintained stable towards Li insertion and extraction during cycling. The excellent redox reversibility in the electrode is also demonstrated by the equal charge calculated by integration of the area under the reductive (discharge) and oxidative (charge) CV peaks. It is worthy to note that the polarization Δ*E* of 50 mV at a sweep rate of 0.1 mV s^−1^ for K_0.03_MnO_2_ NNs is smaller than Δ*E* = 170 mV of α-MnO_2_ synthesized by acid digestion of Mn_2_O_3_ powders, due to the higher electronic conductivity of K-doped MnO_2_ [66] and the fast diffusion kinetics of Li^+^ ions [67]. It was also mentioned that Δ*E* is irrespective of the degree of structural order [27]. Moreover, the peak current *I*_p_ vs. scan rate ν^−1/2^ plot shows a linear behavior, which corresponds to a diffusion-controlled process.

Figure 5b shows the galvanostatic discharge-charge curves of the α-K_0.03_MnO_2_//Li cells cycled in the voltage range of 1.5–4.0 V at constant current density of 30 mA g^−1^ at 25 °C. We can observe a slow decrease of the cell voltage with the presence of two pseudo-plateaus, which are evidenced as an S-shaped profile indicating a topotactic reaction for the Li insertion in the MnO_2_ framework, in which two inequivalent sites are available to coordinate Li^+^. Furthermore, the electrochemical behavior observed in CV measurement is confirmed by GCD experiment. There is a notable difference between the first discharge profile and forthcoming ones with a shift of plateaus to higher potential starting from the second cycle. The material also exhibits a capacity loss in initial four cycles from 211 to 198 mAh g^−1^. The Faraday yield of 0.68 F mol^−1^ for MnO_2_ is consistent with the mean oxidation state +3.885 for Mn ions determined by elemental analysis assuming an equal number of K^+^ and Mn^3+^ cations.

The pronounced changes in the electrochemical profile of the second cycle have been reported several times [20,27,56,68,69,70]. Different hypotheses have been conjectured: (i) A local activation can take place during the initial lithium insertion on the off-center 8*h* and 8*h*’ Wyckoff position (near the walls of the 2 × 2 tunnels), (ii) an ion-exchange reaction inducing a partial extraction of potassium from the centered 4*e* site, and (iii) a fraction of inserted lithium ions become trapped within the crystal structure of MnO_2_ during the first charge that enhances the lattice stabilization. Johnson [71] assigned the increase in voltage plateaus after the first cycle to a decrease in the cell impedance that occurs during cycling of α-MnO_2_ prepared by the acid digestion of Mn_2_O_3_ by H_2_SO_4_ but did not justify this claim by complementary experiments. Considering the small amount of K^+^ ions, the pronounced change in electrochemical behaviors in the second cycle (vs. the first cycle) could be signed to the difference in the site occupation by Li^+^ ions rather than the ion exchange in our sample. Recently, the CV peak shift during first scans has also been observed in the Li-K_y_MnO_2_ system due to the irreversible local activation in the intercalated lattice and the formation of the solid interphase (SEI) layer [27,72].

Figure 5c,d show the incremental capacity curves (IC) (i.e., differential capacity vs. cell voltage (d*Q*/d*V*), of the first and second lithiation processes). This analysis can be considered as an efficient tool to determine the electrochemical spectroscopy of an electrode [73]. For instance, IC has been successfully applied to analyze the behavior of doped or blended cathodes [74,75]. The IC curves were extracted from the GCD profiles (lithiation process) to further characterize the electrochemical behavior at the first and second cycles showing the transformation after the first lithiation process. Each plot displays two broad peaks in the voltage range of 1.5–3.0 V corresponding to the plateaus in GCD curves. These results show clearly the upward potential shift after the first cycle, which indicate a change of the Li location in the host K_y_MnO_2_ framework.

**Figure 6:**
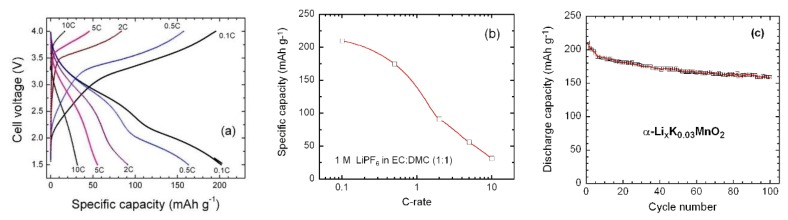
(**a**) Voltage profiles (2nd cycle) of the K_0.03_MnO_2_‖Li cells at various C-rates. (**b**) Modified Peukert plot in the range 0.1 to 10 C. (**c**) Cycling stability of the K_0.03_MnO_2_‖Li cell obtained at 0.1 C.

The rate capability and cycling stability of the α-K_0.03_MnO_2_ electrode were tested in the voltage range 1.5–4.0 V at various current densities in the range 0.1–10 C. Results are presented in Figure 6a–c. Upon the increase of the current density, we observe a decay in the specific capacity without significant change of the charge and discharge profiles (Figure 6a). The S-shaped profile is maintained in the tested C-rate range. As shown in Figure 6b, the modified Peukert plot (i.e., discharge capacity vs. C-rate) exhibits an almost semi-logarithmic behavior, and the α-K_0.03_MnO_2_ electrode delivers a specific capacity of ~32 mAh g^−1^ at 10 C. Figure 6c presents the cycling performance of the α-K_0.03_MnO_2_ electrode carried out at 30 mA g^−1^ up to 100 cycles. Except for the first few cycles, the capacities decrease steadily. The fade rate of 0.3 mAh g^−1^ per cycle for α-K_0.03_MnO_2_ NNs electrode is much smaller than that for the unstabilized α-MnO_2_ (2.3 mAh g^−1^ per cycle) [68]. This behavior is similar to that observed for KMn_8_O_16_ nanofibers [20] and for KMn_8_O_16_ nanorods [26]. The good reversibility of the α-K_0.03_MnO_2_ NNs is evidenced by the relevant coulombic efficiency that remains around 98.8% after several cycles. Considering the satisfying electrochemical stability, it seems that the cationic exchange (i.e., Li^+^ vs. K^+^) is negligible during the Li insertion in the α-K_0.03_MnO_2_ framework.

## 4. Discussion

Although the application of cryptomelane K_y_MnO_2_ nanoparticles have been widely investigated in supercapacitor, to the best our knowledge, there is a limited number of studies devoted as cathode materials in LiBs [20,26,60,72,73,74,75,76,77]. In this work, the nanostructured cryptomelane-type K_y_MnO_2_ has been successfully prepared by the reduction of MnO_4_^−^ ions in acidic medium at room temperature. Nanoneedles are well formed even within a short reaction duration of 1 h. A lot of literature has been reported on the synthesis of nanoparticle α-MnO_2_ based materials, a brief summary is given here. Gao and Norby reported that the growth of α-MnO_2_ nanofibers started after 30 min as a mixture of birnessite and cryptomelane phase via hydrothermal route at 140 °C. Relatively pure cryptomelane nanofibers would only be obtained after a reaction time of ~4 h [37]. Single-crystal α-MnO_2_ nanowires (average diameter of 30 nm) were synthesized by only using potassium permanganate and sodium nitrite (molar KMNO_4_:NaNO_2_ ratio of 2:1) in acidic solution, while nanorods (50–100 nm diameter) were formed for decreasing molar ratio to 2:5 [78]. Similar MnO_2_ nanoneedles with diameter of 10–50 nm and length of 200–500 nm were prepared by a two-step synthesis at temperature of 83 °C with a more complex procedure using MnCl_2_·4H_2_O, isopropanol and KMnO_4_ raw materials [16].

Excellent properties of α-K_y_MnO_2_ have been obtained using a much simple synthesis process, in which the biologic reducing agent allows shorter reaction duration in ambient conditions. Let compare our material with others. Cryptomelane-type K_0_._106_Mn_2_O_4_ nanoneedles were prepared via a template-free, one-step hydrothermal method (HTM) using a complex chemistry composed of manganese(II) acetate tetrahydrate (MnAc_2_4H_2_O) 99%, oxone monopersulfate compound (triple salt 2KHSO_5_KHSO_4_K_2_SO_4_), and potassium nitrate (KNO_3_). Needle-like samples were obtained by HTM at 200 °C for 20 h [38]. Similar process was employed to prepare K_0_._25_Mn_2_O_4_ nanofibers with high potassium content with diameters in the range of 10–20 nm [20]. Liu et al. demonstrated that the size of nanoneedles can be controlled by cross-linking reagents (e.g., polyvinyl acetate (PVA), glycerol and glucose); the shorter K_0.3_MnO_2_ nanocrystals were obtained using PVA [42]. Cheng et al. controlled the synthesis of *α*-MnO_2_ NWs using a hydrothermal method, in which potassium permanganate is reduced by acetic acid. The NWs with a length about 6–10 mm and an average diameter of 20 nm were obtained using 3 mmol KMnO_4_ dissolved in 0.4 mol L^−1^ CH_3_COOH solution (30 mL) [79]. Li et al. succeeded to grow *α*-MnO_2_ nanowires (width of ~30 nm and length of ~10 µm) with exposed (110) crystal plane using KMnO_4_ and NH_4_F in hydrothermal conditions but did not report the presence of tunneled K^+^ ions [80].

The nanoneedles morphology of *α*-K_y_MnO_2_ appeared to be favorable of enhanced electrochemical performance partly due to the modified lattice parameters (i.e., the expansion in the *a*-direction and the slight contraction in the *c*-direction without obvious change in volume). Similar results have been reported by Feng et al. for the urchin-like MnO_2_ formed by the assembly of nanoneedles prepared in acidic conditions [81]. A second key issue is the specific surface area. The BET value of 161 m^2^ g^−1^ is greater than values reported for MnO_2_ prepared by different methods [48,49,50,51,52,53]. BET surface area of 132 m^2^ g^−1^ was reported for mesoporous α-MnO_2_ hollow urchins prepared by mild reaction route [52]. Similar results were reported for mesoporous *α*-MnO_2_ nanorods synthesized by reduction of KMnO_4_ with a triblock polymer assisted by sonication (i.e., a pore volume of 1.0 cm^3^ g^−1^ but smaller pore diameter of 13.5 nm) [23]. Specific surface areas varying in the range of 35–110 m^2^ g^−1^ for *α*-MnO_2_ NWs were shown to be dependent on the growth conditions [21]. *α*-MnO_2_ nanotubes with large surface area (226 m^2^ g^−1^) fabricated by exfoliation of *α*-MnO_2_ nanoflowers exhibited enhanced lithium storage properties [54] The morphology of our sample is different from the heavy K-doped MnO_2_ with fiber-like morphology growing along the *c*-axis [82]. Dai et al. pointed out that only nanoneedles as short fibers are obtained because the weak stabilization of tunnels by low K^+^ ions concentration in the tetragonal crystal [69]. As the growth of *α*-MnO_2_ nanoparticles is time- and temperature-dependent, it is noteworthy that the use of extract of broccoli favors the formation of nanoneedle-shape. During the synthesis, the purple color of KMnO_4_ disappeared quickly after less than 1 h at room temperature and nanoneedles with diameter of about 5 nm and length of about 100 nm were formed. On the contrary, for the synthesis of K_0.08_MnO_2_ from the reduction of KMnO_4_ in sulfuric acid without chelating agent, the final product was obtained after a reaction at high temperature of 60 °C for longer duration of 8 h [53].

Several studies have demonstrated that introduction of K^+^ ions in the cryptomelane structure maintains the integrity of the lattice and is suitable for improving electrochemical properties of α-MnO_2_ when applying as the cathode material for LIBs [37,38,39,72,83]. There is a kind of effective pillaring effect in presence of large cations with an ionic radius of 1.51 Å for eight-coordinated K^+^ ions. Tompsett and Islam performed a comprehensive ab initio study of Li insertion in hollandite α-MnO_2_ but did not consider a lattice with K^+^ ions in the tunnels [61]. However, there is a compromise in the choice of potassium concentration in the 2 × 2 tunnels of MnO_2_. Due to the lattice expansion and the electron donation, higher concentration of K^+^ ions not only improves the Li^+^ diffusion coefficient (*D*_Li_^+^) and enhances the electronic conductivity by boosting electrons hopping via Mn^3+^/Mn^4+^ couples, but also reduces the specific discharge capacity because the addition of inactive K^+^ ions. An enhanced *D*_Li_^+^ has been estimated from 2.8 × 10^−15^ to 1.9 × 10^−12^ cm^2^ s^−1^ when pure *α*-MnO_2_ was doped with 0.25 K^+^ ion per formula [77]. Bach et al. [84] calculated a value of *D*_Li_^+^ ≈ 10^−10^ cm^2^ s^−1^ for the hydrated *α*-phase K_0.062_MnO_2_ using cyclic voltammetry. Recently, using electrochemical impedance spectroscopy, Abuzeid et al. reported *D*_Li_^+^ values of 5.2 × 10^−11^ and 2.1 × 10^−11^ cm^2^ s^−1^ for K_0.11_MnO_2_ and K_0.06_MnO_2_, respectively, showing the diffusivity enhancement with the higher content of K^+^ ions [27]. Meanwhile, different synthetic approaches provide nanostructured cryptomelane-type MnO_2_ with different particle shapes, particle sizes and particle size distributions [85,86]. Additionally, it has been achieved that nanoneedles or nanowires can accommodate large strain without pulverization and exhibit short lithium insertion pathways [87,88].

The *α*-K_y_MnO_2_ nanoneedle-like material reported herein synthesized with the assistance of extract of broccoli plant as reducing agent with a small concentration of potassium has shown excellent electrochemical performances. However, the use of nanoneedles as electrode requested careful fabrication. It is worth noting that the composite hardly adheres to the Al foil during the electrode coating. This may attribute to the rather soft aluminum foil and the weak interaction between the very fine MnO_2_ nanoneedle and aluminum (i.e., van der Waals forces). Thus, the preparation of α-K_y_MnO_2_ electrode with ultra-high mass loading would be difficult and calls for the further study.

It is well known that the discharge capacity of α-K_y_MnO_2_ comes from the insertion of lithium ions on available empty sites inside the (2 × 2) tunnels, i.e., off-center 8*h* Wyckoff positions [61] with the electrochemical reaction of K_y_MnO_2_ + *x*Li^+^ + *x*e^−^ → Li_x_K_y_MnO_2_. However, the real situation seems to be more complex due to the presence of K^+^ ions in the MnO_2_ tunnels. We observed a drastic change of the discharge voltage profile after the first cycle. Such a behavior has been revealed by several reports on *α*-MnO_2_ materials with different tunneled cations [89,90]. After the formation, the voltage plateau remains stable at 2.88 V for our nanoneedle-like samples and is slightly higher than 2.80 V for K_0.00_MnO_2_ reported by Poyraz et al. [39], who showed that the discharge plateau voltage decreases systematically with increasing K^+^ concentration (i.e., 2.62 V for K_0.04_MnO_2_). However, data of the literature are quite controversial showing the major influence of the central cation in the tunnel and the main role of the material morphology. In the early work, Rossouw et al. [91] observed CV curves with two well-resolved steps during cathodic and anodic scans, while Hill et al. [70] revealed a unique broad anodic peak during charge for nanofibers (~15–25 nm in diameter), for which the cathodic peak slightly shifted from 2.82 to 2.90 V. Dai et al. reported also changes in the CV curves in cryptomelane-type hydronium-doped MnO_2_ (i.e., (H_3_O)_0.106_MnO_2_·0.3H_2_O) upon Li insertion that implies similar free energies for the different hosting Li sites after several cycles [68]. Johnson et al. [89] indicated the disappearance of the voltage plateau-type shape at about 2.5 V for lithia-stabilized *α*-MnO_2_ (i.e., *α*-(Li_2_O)_0.143_MnO_2_), while the discharge profile change is less pronounced for ammonia-stabilized sample (i.e., α-(NH_3_)_0.2_MnO_2_). Kijima et al. confirmed the loss of the plateau with cycling of *α*-(Li_2_O)_0.12_MnO_2_) [90]. This group demonstrated initial discharge capacity improvement by stabilizing the structure with chemical insertion of two Li-ions per unit cell. Zhang et al. reported the electrochemical performance of *α*-K_1.0_Mn_8_O_16_ synthesized by hydrothermal route at 120 °C that showed a voltage plateau at ca. 2.3 V and an initial discharge capacity of 360 mAh g^−1^ at current density of 0.1 A g^−1^. A non-negligible amount of K^+^ ions were extracted from the K_1.0_Mn_8_O_16_ nanofibers cathode in the first charge reaction but showed a Coulombic efficiency of ~50% at C/3 rate [20]. Similar conclusion was claimed for the electrochemical reactions KMn_8_O_16_ nanorods [26]. In contrast, Ranjusha et al. [60] observed GCD single slopped curve without distinct voltage plateau for well-crystallized MnO_2_ nanowires (30 nm in diameter) prepared by oxidation of Mn^2+^ by MnO_4_^−^ and an initial capacity of 251 mAh g^−1^ at C/2 rate but the K^+^ content was not mentioned. Kumakai et al. demonstrated a two S-shaped discharge behavior with plateaus at 2.6 and 2.3 V for K_0.72_Mn_8_O_16_ prepared by hydrothermal method at 80 °C [76]. They stated that, in *α*-K_y_MnO_2_ and its Co-doped specimen (K_y_Mn_0.88_Co_0.12_O_2_), the Li is inserted into two types of empty sites of the small (1 × 1) and large (2 × 2) tunnels with an initial discharge capacity of 250 mAh g^−1^ at 10 mA g^−1^.

Table 1 compares the electrochemical performance of the nanoneedle-like α-K_y_MnO_2_ prepared in this work to previous reported cryptomelane-type *α*-K_y_MnO_2_ cathode materials. In the early work by Ohzuku et al. [92], K_y_Mn_8_O_16_ cathode materials prepared by two different methods showed S-shaped discharge curves with the relatively high voltage plateaus at 3.0 and 2.45 V. Sol–gel K_1.2_Mn_8_O_16_ and acid digestion K_1.3_Mn_8_O_16_ exhibit modest initial discharge capacities of 160 and 188 mAh g^−1^, when cycled at 50 mA g^−1^, respectively, due to the micrometer-sized particles [91]. Similar results were obtained for heavy doped *α*-K_y_Mn_8_O_16_ nanorods (*y* ≈1, with a diameter of 5–15 nm and a length of 50–300 nm) synthesized using a reflux method with capacity of 159 mAh g^−1^ at 50 mA g^−1^ [26]. A comparison between K_0.25_MnO_2_ prepared by hydrothermal method at 160 °C and MnO_2_ obtained by acid digestion (HNO_3_ treatment) of the K-doped material shows specific discharge capacities of 143 and 170 mAh g^−1^ at current density of 0.1 A g^−1^, while in terms of cyclability, the capacity retention of 62% at 5 C rate is better for K_0.25_MnO_2_ than for the undoped cathode [74]. The Co-doped hydrothermal product K_0.14_Mn_0.9_Co_0.1_O_2_ from MnSO_4_ and K_2_S_2_O_8_ solution showed higher initial discharge capacity 180–200 mAh g^−1^ at 50 mA g^−1^ [93]. After 200 cycles the hydrated K_0.84_Mn_8_O_16_·0.25H_2_O nanowire-type electrode material fabricated by thermal regeneration process of MnO_2_ waste delivered a specific capacity of 120 mAh g^−1^ at 50 mA g^−1^ [94]. K_y_MnO_2_ nanorods (100 nm in size) prepared by template-based sol-gel method delivered an initial discharge capacity of 183 mAh g^−1^, which stabilized on subsequent cycles to 134 mAh g^−1^ [95]. Urchin-like α-K_0.04_MnO_2_ formed by nanoneedles synthesized by sol-gel assisted by a redox reaction between ascorbic acid and KMnO_4_ showed an outstanding initial specific capacity of 230 mAh g^−1^ and 45% capacity retention at cycle one hundred [96]. Compared to the state-of-art of MDO materials, the cryptomelane-type *α*-K_y_MnO_2_ cathode prepared via biological reducing agent (broccoli extract) assisted synthesis shows good electrochemical performance.

## 5. Conclusions

In this work, one-dimensional α-K_y_MnO_2_ nanoneedles in pure cryptomelane phase were successfully prepared by a facile, one pot, scalable and environmentally-friendly method. An alternative biological reducing agent, i.e., extract of broccoli plant, was used instead of a traditional chemical chelate. This green-synthesis route is a straightforward and inexpensive facilitating mass production of nanostructured *α*-MnO_2_ particles stabilized by potassium ions without templates. The nanostructure characterized by crystallite of 4.4 nm, large surface area 160.7 m^2^ g^−1^, high porosity and low potassium content of 0.03 per formula in the 2 × 2 tunnels favors good electrochemical performance. Electrochemical tests show an initial capacity ca. 211 mAh g^−1^ with high Coulombic efficiency of 99% and good reversibility with a capacity retention 82% after 100 cycles. Thus, α-K_0.03_MnO_2_ nanoneedle-shaped material synthesized via the broccoli-extract-assisted green process could be considered as a promising cathode for LiBs.

## Figures and Tables

**Table 1:** Comparison of electrochemical performance of cryptomelane-type *α*-K_y_MnO_2_ cathode materials.

Composition	Morphology (Synthesis) ^a^	Specific Capacity	Current Density	
		(mAh g^−1^)	(mA g^−1^)	Ref.
K_y_MnO_2_	NRs (H)	189	50	[68]
K_0.06_MnO_2_	NNs (R)	236	10	[27]
K_0.11_MnO_2_	NNs (H)	198	10	[27]
K_0.14_MnO_2_	NNs (H)	160	50	[93]
K_0.14_Mn_0.9_Co_0.1_O_2_	NNs (H)	200	50	[93]
K_0.25_MnO_2_	NWs (H)	143	100	[77]
K_0.125_MnO_2_	NRs (Ox)	160	50	[26]
K_0.84_Mn_8_O_16_·0.25H_2_O	NWs (Rc)	120	50	[94]
K_y_MnO_2_	NRs (R)	183	10	[95]
K_0.04_MnO_2_	NNs (R)	230	30	[96]
K_0.32_Mn_8_O_16_	NFs (H)	200	50	[39]
K_0.75_Mn_8_O_16_	NFs (H)	165	50	[39]
K_0.25_Mn_8_O_16_	NKs (R)	260	50	[67]
K_0.125_MnO_2_	NFs (H)	190	100	[20]
K_0.03_MnO_2_	NNs (R)	210	30	this work

^a^ H—hydrothermal; R—redox reaction; Ox—oxidation of Mn^2+^; Rc—MnO_2_ recycling.

## References

[1] Betz J., Bieker G., Meister P., Placke T., Winter M., Schmuch R. (2019). Theoretical vs. practical energy: A plea for more transparency in the energy calculation of different rechargeable battery systems. Adv. Energy Mater..

[2] Winter M., Brodd R.J. (2004). What are batteries, fuel cells and supercapacitors?. Chem. Rev..

[3] Julien C.M., Mauger A., Vijh A., Zaghib K. (2016). Lithium Batteries: Science and Technology.

[4] Fergus J.W. (2010). Recent developments in cathode materials for lithium ion batteries. J. Power Sources.

[5] Jiao F., Bruce P.G. (2007). Mesoporous crystalline β-MnO_2_–a reversible positive electrode for rechargeable lithium batteries. Adv. Mater..

[6] GlobTeck, Inc. Lithium Manganese Dioxide Battery Li-MnO_2_. https://fr.globtek.com/lithium-manganese-dioxide-battery-li-mno2-batteries/.

[7] Zhang K., Han X., Hu Z., Zhang X., Tao Z., Chen J. (2015). Nanostructured Mn-based oxides for electrochemical energy storage and conversion. Chem. Soc. Rev..

[8] Reddy A.L.M., Shaijumon M.M., Gowda S.R., Ajayan P.M. (2009). Coaxial MnO_2_/carbon nanotube array electrodes for high-performance lithium batteries. Nano Lett..

[9] Julien C.M., Mauger A. (2017). Nanostructured MnO_2_ as electrodes materials for energy storage. Nanomaterials.

[10] Tang Y., Zheng S., Xu Y., Xiao X., Xue H., Pang H. (2018). Advanced batteries based on manganese dioxide and its composites. Energy Storage Mater..

[11] He X., Wang J., Jia H., Kloepsch R., Liu H., Beltrop K., Li J. (2015). Ionic liquid-assisted solvothermal synthesis of hollow Mn_2_O_3_ anode and LiMn_2_O_4_ cathode materials for Li-ion batteries. J. Power Sources.

[12] Li Z., Zhang J., Lou X.W. (2015). Hollow carbon nanofibers filled with MnO_2_ nanosheets as efficient sulfur hosts for lithium-sulfur batteries. Angew. Chem. Int. Ed..

[13] Débart A., Peterson A.J., Bao J., Bruce P.G. (2008). α-MnO_2_ nanowires: A catalyst for the O_2_ electrode in rechargeable lithium batteries. Angew. Chem. Int. Ed..

[14] Toupin M., Brousse T., Bélanger D. (2004). Charge storage mechanism of MnO_2_ electrode used in aqueous electrochemical capacitor. Chem. Mater..

[15] Kim M., Hwang Y., Min K., Kim J. (2013). Introduction of MnO_2_ nanoneedles to activated carbon to fabricate high-performance electrodes as electrochemical supercapacitors. Electrochim. Acta.

[16] Chen S., Zhu J., Han Q., Zheng Z., Yang Y., Wang X. (2009). Shape-controlled synthesis of one-dimensional MnO_2_ via a facile quick-precipitation procedure and its electrochemical properties. Cryst. Growth Des..

[17] Zeng Y., Zhang X., Meng Y., Yu M., Yi J., Wu Y., Lu X., Tong Y. (2017). Achieving ultrahigh energy density and long durability in a flexible rechargeable quasi-solid-state Zn-MnO_2_ battery. Adv. Mater..

[18] Housel L.M., Wang L., Abraham A., Huang J., Renderos G.D., Quilty C.D., Brady A.B., Marschilok A.C., Takeuchi K.J., Takeuchi E.S. (2018). Investigation of α-MnO_2_ tunneled structures as model cation hosts for energy storage. Acc. Chem. Res..

[19] Vicat J., Fanchon E., Strobel P., Tran-Qui D. (1986). The structure of K_1.33_Mn_8_O_16_ and cation ordering in hollandite-type structures. Acta Crystallogr. B.

[20] Zhang C., Feng C., Zhang P., Guo Z., Chen Z., Lid S., Liu H. (2012). K_0_._25_Mn_2_O_4_ nanofiber microclusters as high power cathode materials for rechargeable lithium batteries. RSC Adv..

[21] Portehault D., Cassaignon S., Baudrin E., Jolivet J.P. (2007). Morphology control of cryptomelane type MnO_2_ nanowires by soft chemistry. Growth mechanisms in aqueous medium. Chem. Mater..

[22] Li L., Pan Y., Chen L., Li G. (2007). One-dimensional α-MnO_2_: Trapping chemistry of tunnel structures, structure stability, and magnetic transitions. J. Solid State Chem..

[23] Nayak P.K., Munichandraiah N. (2012). Rapid sonochemical synthesis of mesoporous MnO_2_ for supercapacitor applications. Mater. Sci. Eng. B.

[24] Devaraj S., Munichandraiah N. (2007). Electrochemical supercapacitor studies of nanostructured *α*-MnO_2_ synthesized by microemulsion method and the effect of annealing. J. Electrochem. Soc..

[25] Abuzeid H.M., Elsherif S.A., Abdel-Ghany N.A., Hashem A.M. (2019). Facile, cost-effective and eco-friendly green synthesis method of MnO_2_ as storage electrode materials for supercapacitors. J. Energy Storage.

[26] Zheng H., Feng C., Kim S.-J., Yin S., Wu H., Wang S., Li S. (2013). Synthesis and electrochemical properties of KMn_8_O_16_ nanorods for lithium ion batteries. Electrochim. Acta.

[27] Abuzeid H.M., Hashem A.M., Kaus M., Knapp M., Indris S., Ehrenberg H., Mauger A., Julien C.M. (2018). Electrochemical performance of nanosized MnO_2_ synthesized by redox route using biological reducing agents. J. Alloys Compd..

[28] Porrawatkul P., Nuthong W., Pimsen R., Thongsom M. (2017). Green synthesis of silver nanoparticles using *Barringtonia acutangula* (L.) Gaertn leaf extract as reducing agent and their antibacterial and antioxidant activity. J. Appl. Sci..

[29] Fatimah I. (2016). Green synthesis of silver nanoparticles using extract of Parkia speciose Hassk pods assisted by microwave irradiation. J. Adv. Res..

[30] Cartea M.E., Francisco M., Soengas P., Velasco P. (2011). Phenolic compounds in brassica vegetables. Molecules.

[31] Hashem A.M., Abuzeid H., Kaus M., Indris S., Ehrenberg H., Mauger A., Julien C.M. (2018). Green synthesis of nanosized manganese dioxide as positive electrode for lithium-ion batteries using lemon juice and citrus peel. Electrochim. Acta.

[32] Kochhar M., Kochhar A. (2014). Proximate composition, available carbohydrates, dietary fibre and anti-nutritional factors of broccoli (*Brassica oleracea* L. Var. Italica Plenck) leaf and floret powder. Biosci. Discov..

[33] Campas-Baypoli O.N., Sánchez-Machado D.I., Bueno-Solano C., Núñez-Gastélum J.A., Reyes-Moreno C., López-Cervantes J. (2009). Biochemical composition and physicochemical properties of broccoli flours. Int. J. Food Sci. Nutr..

[34] Vallejo F., Tomas-Barberan F., Garcia-Viguera C. (2003). Health-promoting compounds in broccoli as influenced by refrigerated transport and retail sale period. J. Agric. Food Chem..

[35] Pathare P.B., Mohapatra D., Kadam D.M., Sharma M., Kaur D. (2017). Bioactive compounds in broccoli: Extraction and processing. Vegetable Processing and Bioactive Compounds.

[36] Barrett E.P., Joyner L.G., Halenda P.P. (1951). The determination of pore volume and area distributions in porous substances. I. Computations from nitrogen isotherms. J. Am. Chem. Soc..

[37] Gao T., Norby P. (2013). Frame stability of tunnel-structured cryptomelane nanofibers: The role of tunnel cations. Eur. J. Inorg. Chem..

[38] Galindo H.M., Carvajal Y., Njagi E., Ristau R.A., Suib S.L. (2010). Facile one-step template-free synthesis of uniform hollow microstructures of cryptomelane-type manganese oxide K-OMS-2. Langmuir.

[39] Poyraz A.S., Huang J., Pelliccione C.J., Tong X., Cheng S., Wu L., Zhu Y., Marschilok A.C., Takeuchi K.J., Takeuchi E.S. (2017). Synthesis of cryptomelane type α-MnO_2_ (K_x_Mn_8_O_16_) cathode materials with tunable K^+^ content: The role of tunnel cation concentration on electrochemistry. J. Mater. Chem. A.

[40] Gaillot A.-C., Flot D., Drits V.A., Manceau A., Burghammer M., Lanson B. (2003). Structure of synthetic K-rich birnessite obtained by high-temperature decomposition of KMnO_4_. I. Two-layer polytype from 800 °C experiment. Chem. Mater..

[41] Wang X., Li Y. (2003). Synthesis and formation mechanism of manganese dioxide nanowires/nanorods. Chem. Eur. J..

[42] Liu J., Son Y.C., Cai J., Shen X., Suib S.L., Aindow M. (2004). Size control, metal substitution, and catalytic application of cryptomelane nanomaterials prepared using cross-linking reagents. Chem. Mater..

[43] McKenzie R.M. (1971). The synthesis of birnessite, cryptomelane and some other oxides and hydroxides of manganese. Miner. Mag..

[44] Davoglio R.A., Cabello G., Marco J.F., Biaggio S.R. (2018). Synthesis and characterization of α-MnO_2_ nanoneedles for electrochemical supercapacitors. Electrochim. Acta.

[45] Villegas J.C., Garces L.J., Gomez S., Durand J.P., Suib S.L. (2005). Particle size control of cryptomelane nanomaterials by use of H_2_O_2_ in acidic conditions. Chem. Mater..

[46] Kumar V.G., Kim K.B. (2006). Organized and highly dispersed growth of MnO_2_ nano-rods by sonochemical hydrolysis of Mn(3)acetate. Ultrason. Sonochem..

[47] Poyraz A.S., Kuo C.-H., Biswas S., King’ondu C.K., Suib S.L. (2013). A general approach to crystalline and monomodal pore size mesoporous materials. Nat. Commun..

[48] Xia A., Yu W., Tan G., Ren H., Liu C. (2019). Synthesis of porous δ-MnO_2_ nanosheets and their supercapacitor performance. J. Electroanal. Chem..

[49] Li L., Nan C., Lu J., Peng Q., Li Y. (2012). α-MnO_2_ nanotubes: High surface area and enhanced lithium battery properties. Chem. Commun..

[50] Devaraj S., Munichandraiah N. (2008). Effect of crystallographic structure of MnO_2_ on its electrochemical capacitance properties. J. Phys. Chem. C.

[51] Subramanian V., Zhu H., Vajtai R., Ajayan P.M., Wei B. (2005). Hydrothermal synthesis and pseudocapacitance properties of MnO_2_ nanostructures. J. Phys. Chem. B.

[52] Chen H., Dong X., Shi J., Zhao J., Hua Z., Gao J., Ruan M., Yan D. (2007). Templated synthesis of hierarchically porous manganese oxide with a crystalline nanorod framework and its high electrochemical performance. J. Mater. Chem..

[53] Li B., Rong G., Xie Y., Huang L., Feng C. (2006). Low-temperature synthesis of alpha-MnO_2_ hollow urchins and their application in rechargeable Li^+^ batteries. Inorg. Chem..

[54] Zhang X., Yu P., Zhang H., Zhang D., Sun X., Ma Y. (2013). Rapid hydrothermal synthesis of hierarchical nanostructures assembled from ultrathin birnessite-type MnO_2_ nanosheets for supercapacitor applications. Electrochim. Acta.

[55] Alfaruqi M.H., Islam S., Gim J., Song J., Kim S., Pham D.T., Jo J., Xiu Z., Mathew V., Kim J. (2016). A high surface area tunnel-type α-MnO_2_ nanorod cathode by a simple solvent-free synthesis for rechargeable aqueous zinc-ion batteries. Chem. Phys. Lett..

[56] Yuan Y., Nie A., Odegard G.M., Xu R., Zhou D., Santhanagopalan S., He K., Asayesh-Ardakani H., Meng D.D., Klie R.F. (2015). Asynchronous crystal cell expansion during lithiation of K^+^-stabilized α-MnO_2_. Nano Lett..

[57] Ohzuku T., Tari I., Hirai T. (1982). Thermal gravimetric studies of manganese dioxide. Electrochim. Acta.

[58] Muraoka Y., Chiba H., Atou T., Kikuchi M., Hiraga K., Syono Y., Sugiyama S., Yamamoto S., Grenier J.C. (1999). Preparation of α-MnO_2_ with an open tunnel. J. Solid State Chem..

[59] Feng Q., Kanoh H., Miyai Y., Ooi K. (1995). Alkali metal ions insertion/extraction reactions with hollandite-type manganese oxide in the aqueous phase. Chem. Mater..

[60] Ranjusha R., Sonia T.S., Roshny S., Lakshmi V., Kalluri S., Kim T.N., Nair S.V., Balakrishnan A. (2015). Synthesis, characterization and rate capability performance of the micro-porous MnO_2_ nanowires as cathode material in lithium batteries. Mater. Res. Bull..

[61] Sarasketa-Zabala E., Aguesse F., Villareal I., Rodriguez-Martinez L.M., Lopez C.M., Kubiak P. (2015). Understanding lithium inventory loss and sudden performance fade in cylindrical cells during cycling with deep-discharge steps. J. Phys. Chem. C.

[62] Tompsett D.A., Islam M.S. (2013). Electrochemistry of hollandite a-MnO_2_: Li-ion and Na-ion insertion and Li_2_O incorporation. Chem. Mater..

[63] Esmanski A., Ozin G.A. (2009). Silicon inverse-opal-based microporous materials as negative electrodes for lithium ion batteries. Adv. Funct. Mater..

[64] Chan C.K., Peng H., Liu G., McIlwrath K., Zhang X.F., Huggins R.A., Cui Y. (2008). High-performance lithium battery anodes using silicon nanowires. Nat. Nanotechnol..

[65] Wang L., Deng M., Ding G., Chen S., Xu F. (2013). Manganese dioxide based ternary nanocomposite for catalytic reduction and nonenzymatic sensing of hydrogen peroxide. Electrochim. Acta.

[66] Johnson C.S., Dees D.W., Mansuetto M.F., Thackeray M.M., Vissers D.R., Argyriou D., Loong C.K., Christensen L.J. (1997). Structural and electrochemical studies of α-manganese dioxide (α-MnO_2_). J. Power Sources.

[67] Tseng L.-T., Lu Y., Fan H.M., Wang Y., Luo X., Liu T., Munroe P., Li S., Yi J. (2015). Magnetic properties in α-MnO_2_ doped with alkaline elements. Sci. Rep..

[68] Yang Y., Xiao L., Zhao Y., Wang F. (2008). Hydrothermal synthesis and electrochemical characterization of α-MnO_2_ nanorods as cathode material for lithium batteries. Int. J. Electrochem. Sci..

[69] Dai J., Li S.F.Y., Siow K.S., Gao Z. (2000). Synthesis and characterization of the hollandite-type MnO_2_ as a cathode material in lithium batteries. Electrochim. Acta.

[70] Hill L.I., Verbaere A., Guyomard D. (2003). MnO_2_ (α-, β-, γ-) compounds prepared by hydrothermal-electrochemical synthesis: Characterization, morphology, and lithium insertion behavior. J. Power Sources.

[71] Johnson C.S. (2007). Development and utility of manganese oxides as cathodes in lithium batteries. J. Power Sources.

[72] Kim K., Daniel G., Kessler V.G., Seisenbaeva G.A., Pol V.G. (2018). Basic medium heterogeneous solution synthesis of α-MnO_2_ nanoflakes as an anode or cathode in half cell configuration (vs. lithium) of Li-ion batteries. Nanomaterials.

[73] Zhang X., Jiang W.J., Mauger A., Gendron F., Julien C.M., Qilu R. (2010). Minimization of the cation mixing in Li_1+x_(NMC)_1-x_O_2_ as cathode material. J. Power Sources.

[74] Kobayashi T., Kawasaki N., Kobayashi Y., Shono K., Mita Y., Miyashiro H. (2014). A method of separating the capacities of layer and spinel compounds in blended cathode. J. Power Sources.

[75] Hashem A.M., Abdel-Ghany A.E., Scheuermann M., Indris S., Ehrenberg H., Mauger A., Julien C.M. (2019). Doped nanoscale NMC333 as cathode materials for Li-ion batteries. Materials.

[76] Kumagai N., Sasaki T., Oshitari S., Komaba S. (2006). Characterization and lithium insertion characteristics of hollandite-type K_y_(Mn_1-x_M_x_)O_2_ for rechargeable lithium battery electrodes. J. New Mater. Electrochem. Syst..

[77] Yuan Y., Zhan C., He K., Chen H., Yao W., Shari-Asl S., Song B., Yang Z., Nie A., Luo X. (2016). The influence of large cations on the electrochemical properties of tunnel-structured metal oxides. Nat. Commun..

[78] Kumar N., Dineshkumar P., Rameshbabu R., Sen A. (2015). Morphological analysis of ultra fine α-MnO_2_ nanowires under different reaction conditions. Mater. Lett..

[79] Cheng G., Yu L., Lan B., Sun M., Lin T., Fu Z., Su X., Qiu M., Guo C., Xu B. (2016). Controlled synthesis of α-MnO_2_ nanowires and their catalytic performance for toluene combustion. Mater. Res. Bull..

[80] Li W., Cui X., Zeng R., Du G., Sun Z., Zheng R., Ringer S.P., Dou S.X. (2015). Performance modulation of *α*-MnO_2_ nanowires by crystal facet engineering. Sci. Rep..

[81] Feng L., Xuan Z., Zhao H., Bai Y., Guo J., Su C., Chen X. (2014). MnO_2_ prepared by hydrothermal method and electrochemical performance as anode for lithium-ion battery. Nanoscale Res. Lett..

[82] Xiao T.D., Bokhimi X., Benaissa M., Perez R., Strutt P.R., Yacaman M.J. (1997). Microstructural characteristics of chemically processed manganese oxide nanofibers. Acta Mater..

[83] Ahn D., Yoo I., Koo Y.-M., Shin N., Kim J., Shin T.J. (2011). Effects of cobalt-intercalation and polyaniline coating on electrochemical performance of layered manganese oxides. J. Mater. Chem..

[84] Bach S., Pereira-Ramos J.P., Baffier N. (1995). A new MnO2 tunnel related phase as host lattice for Li intercalation. Solid State Ion..

[85] Huang H., Sithambaram S., Chen C.-H., Kithongo C.K., Xu L., Iyer A., Garces H.F., Suib S.L. (2010). Microwave-assisted hydrothermal synthesis of cryptomelane-type octahedral molecular sieves (OMS-2) and their catalytic studies. Chem. Mater..

[86] Hu B., Chen C.-H., Frueh S.J., Jin L., Joesten R., Suib S.L. (2010). Removal of aqueous phenol by adsorption and oxidation with doped hydrophobic cryptomelane-type manganese oxide (K−OMS-2) nanofibers. J. Phys. Chem. C.

[87] Green M., Fielder E., Scrosati B., Wachtler M., Moreno J.S. (2003). Structured silicon anodes for lithium battery applications. Electrochem. Solid-State Lett..

[88] Cheng F.-Y., Zhao J.-Z., Song W., Li C.-S., Ma H., Chen J., Shen P.-W. (2006). Facile controlled synthesis of MnO_2_ nanostructures of novel shapes and their application in batteries. Inorg. Chem..

[89] Johnson C.S., Thackeray M.M. (2001). Ammonia- and lithia-doped manganese dioxide for 3 V lithium batteries. J. Power Sources.

[90] Kijima N., Takahashi Y., Akimoto J., Awaka J. (2005). Lithium ion insertion and extraction reactions with hollandite-type manganese dioxide free from any stabilizing cations in its tunnel cavity. J. Solid State Chem..

[91] Rossouw M.H., Liles D.C., Thackeray M.M. (1992). Alpha manganese dioxide for lithium batteries: A structural and electrochemical study. Mater. Res. Bull..

[92] Ohzuku T., Kitagawa M., Sawai K., Hirai T. (1991). Topotactic reduction of alpha-manganese (di)oxide in nonaqueous lithium cells. J. Electrochem. Soc..

[93] Kadoma Y., Akahira T., Fukuda T., Ui K., Kumagai N. (2012). Synthesis and electrochemical properties of nanofiber hollandite-type manganese oxides using hydrothermal method. Funct. Mater. Lett..

[94] Poyraz A.S., Huang J., Cheng S., Bock D.C., Wu L., Zhu Y., Marschilok A.C., Takeuchi K.J., Takeuchi E.S. (2016). Effective recycling of manganese oxide cathodes for lithium based batteries. Green Chem..

[95] Sugantha M., Ramakrishnan P.A., Hermann A.M., Warmsingh C.P., Ginley D.S. (2003). Nanostructured MnO_2_ for Li batteries. Int. J. Hydrog. Energy.

[96] Hashem A.M., Abdel-Ghany A.E., El-Tawil R., Bhaskar A., Hunzinger B., Ehrenberg H., Mauger A., Julien C.M. (2016). Urchin-like α-MnO_2_ formed by nanoneedles for high-performance lithium batteries. Ionics.

